# Dupilumab, a Potential Novel Treatment for Hailey–Hailey Disease

**DOI:** 10.3390/clinpract15030048

**Published:** 2025-02-26

**Authors:** Liliana Gabriela Popa, Calin Giurcaneanu, Florentina Zaharia, Andreea Grigoras, Alexandra Denisa Oprea, Cristina Beiu

**Affiliations:** 1 Dermatology Department, Elias Emergency University Hospital, 17 Marasti Bd., District 1, 011461 Bucharest, Romaniaalexandra-denisa.oprea@rez.umfcd.ro (A.D.O.);; 2 Dermatology Department, Carol Davila University of Medicine and Pharmacy, 37 Dionisie Lupu Street, District 1, 020021 Bucharest, Romania; 3 Dermatology Department, CF 2 Clinical Hospital, 63 Marasti Bd., District 1, 011464 Bucharest, Romania

**Keywords:** Hailey–Hailey disease, benign familial pemphigus, benign chronic pemphigus, dupilumab

## Abstract

**Background/Objectives:** Hailey–Hailey disease (HHD) is an uncommon genodermatosis with autosomal dominant inheritance caused by loss-of-function mutations in the ATP2C1 gene, which lead to disruption in keratinocyte adhesion and intraepidermal acantholysis. The chronic nature of the disease, its frequent recurrences and the lack of specific treatment pose real challenges in the long-term management of these patients. Recent studies have evaluated the effect of dupilumab, a human monoclonal antibody that blocks interleukin-4 and -13 receptor in refractory HHD, with very promising results. The aim of this study was to review the published data on the use of dupilumab for the treatment of HHD, to present our own experience in the field, and to discuss the mechanisms underlying dupilumab’s beneficial effects in HHD and the future treatment perspectives. **Methods:** A search of the medical literature on the use of dupilumab in the treatment of HHD was conducted. The terms “Hailey–Hailey disease”, “benign familial pemphigus”, “benign chronic pemphigus”, and “dupilumab” were searched across multiple databases (Medline, Chrocane Library, EMBASE) from inception until 30 September 2024. **Results:** To date, six manuscripts describing 11 refractory HHD cases treated with dupilumab have been published. All the patients experienced significant clinical improvement. The authors reported sustained disease quiescence in seven patients (64%), monitored for 5 to 24 months. None of the patients experienced adverse effects related to dupilumab. To the existing evidence, we add a new case of recalcitrant HHD successfully treated with dupilumab. **Conclusions:** Mounting evidence indicates dupilumab as a safe and efficient therapeutic alternative in patients with severe, refractory HHD. However, the long-term efficacy of dupilumab and the optimal therapeutic regimen for HHD are yet to be determined.

## 1. Introduction

Hailey–Hailey disease (HHD), also known as benign familial pemphigus or benign chronic pemphigus, is an uncommon genodermatosis with autosomal dominant inheritance characterized by disruption in keratinocyte adhesion and consequent development of intraepidermal acantholysis [1]. It is caused by loss-of-function variants in the adenosine triphosphatase (ATPase) secretory pathway Ca^2+^ transporting 1 (ATP2C1) gene located on chromosome 3q 21–24 that lead to defective Golgi apparatus calcium homeostasis [2].

The prevalence of HHD is estimated at approximately 1 in 50,000 individuals [1]. Two peaks of incidence have been described, one in late adolescence and the other in the third and fourth decades of life [3]. HHD shows no gender or ethnic predilection [4]. Although it is an autosomal dominant disease with complete penetrance, the age of onset, clinical presentation, and disease severity vary widely between individuals, even within the same family [5].

As a result of intraepidermal acantholysis, vesicles, bullae, erosions, and maceration develop in a chronic and recurrent manner, predominantly affecting intertriginous areas. The scalp, antecubital and popliteal fossae, and the genital region are less commonly interested. The palms, soles, and mucous membranes are rarely involved [5]. Generally, the initial skin lesions are flaccid bullae that arise on an erythematous base. The blisters rupture easily, progressing to eczematous plaques with multiple fissures and crusting. Additionally, vesiculo-pustular and erosive circinate lesions with central hyperchromia may be observed. The lesions are typically symmetric, although case reports of asymmetric and segmental HHD have also been published [6,7]. Chronic lesions may develop into vegetative or verrucous plaques. While postinflammatory hyperpigmentation is common, scarring does not usually occur [4]. Symptoms such as pruritus, burning sensation, and pain can significantly impact the patients’ quality of life. Chronic pruritus is challenging to manage, adding significantly to the disease burden. Due to skin maceration and superinfection of the lesions, the affected areas may develop a foul odor [8]. Nail alterations, present in up to 70% of cases, are especially represented by longitudinal leukonychia [4]. The course of the disease is frequently marked by relapses, usually triggered by trauma and friction, exposure to sunlight or a hot, humid environment, or by certain drugs, including nonsteroidal anti-inflammatory medication and efalizumab [7,9,10]. The lesions are often complicated by bacterial, fungal, or viral superinfection [3,4,5]. Exacerbations during pregnancy and menstruation have also been described [7]. A rare but severe complication of longstanding active lesions is malignant degeneration into squamous cell carcinoma [11].

The diagnosis is usually straightforward, given the patient’s family history and the characteristic clinical and histopathological features [4]. However, HHD may mimic common dermatoses, including eczema, tinea, erythrasma, intertrigo, and inverse psoriasis, as well as less common conditions, such as Darier disease, Galli–Galli disease, granular parakeratosis, and pemphigus vegetans, delaying the correct diagnosis. The clinical suspicion is confirmed by histopathological examination, which reveals extensive, often full-thickness epidermal acantholysis with minimal or no dyskeratosis. Sometimes, the keratinocytes remain connected through adherens junctions. This pattern creates a characteristic histological appearance, compared to “a dilapidated brick wall” [8]. Parakeratosis, epidermal hyperplasia, and a dermal perivascular lymphocytic inflammatory infiltrate are also present [12]. Immunofluorescence tests are negative.

**Figure 1:**
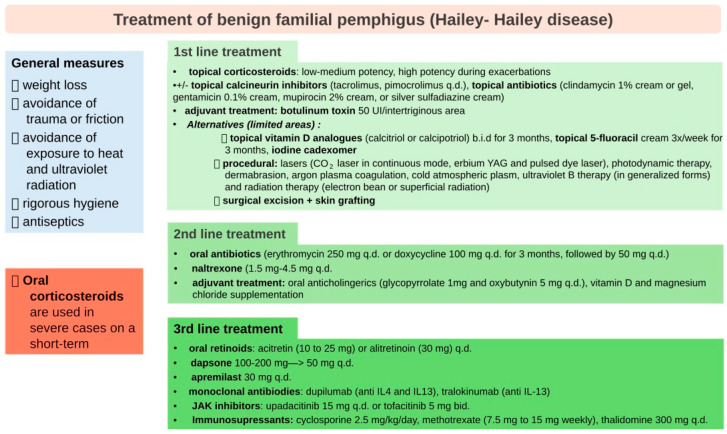
Treatment of benign familial pemphigus (Hailey–Hailey diseases).

The chronic nature of the disease, its frequent recurrences, and the lack of specific treatment pose real challenges in the long-term management of these patients. Due to the rarity of HHD, no randomized controlled trials have been conducted in order to determine the optimal therapeutic approach [8]. Compliance with general measures, such as weight loss, avoidance of trauma or friction in the affected areas and exposure to heat and ultraviolet radiation, wearing loose clothes and absorbent pads in intertriginous areas, and maintaining a rigorous hygiene is essential for preventing exacerbations. Topical corticosteroids, calcineurin inhibitors, vitamin D analogs, and antibiotics/antifungals are used to reduce inflammation and prevent infection. Conventional systemic treatments for HHD are mainly represented by oral antibiotics, retinoids, and corticosteroids. In severe cases, immunosuppressive agents may be considered [4]. Various procedures, such as botulinum toxin type A injections, laser treatment, photodynamic therapy, radiofrequency surgery, dermabrasion, and surgical excision of the affected area, as well as superficial radiation and electron beam radiation have been performed in recalcitrant HHD cases, with variable efficacy (Figure 1) [7]. Recent studies have also evaluated the effect of dupilumab, a human immunoglobulin (Ig) G4 monoclonal antibody that blocks interleukin (IL)-4 and -13 receptor in refractory HHD, with very promising results [13,14,15,16,17,18].

The aim of this study is to review the published data on the use of dupilumab for the treatment of HHD, to present our own experience in the field, and to discuss the mechanisms underlying dupilumab’s beneficial effects in HHD and the future treatment perspectives.

## 2. Materials and Methods

### Systematic Review of the Literature

**Figure 2:**
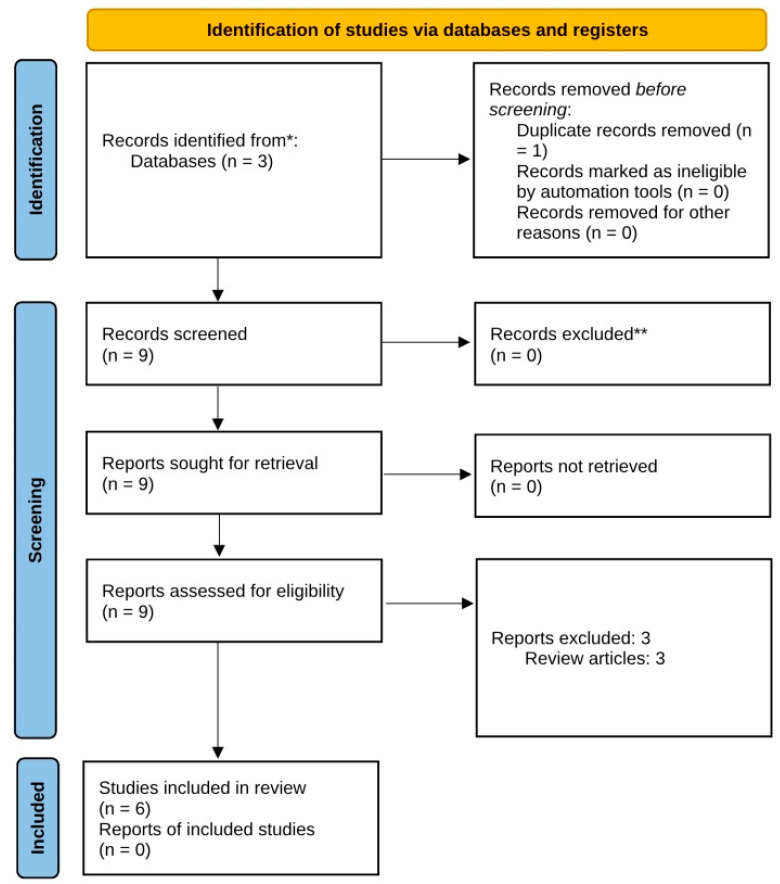
Prisma flow chart. *: Medline, Chrocane Library, EMBASE; **: all records were independently assessed by two authors, no automation tools were used.

In order to better evaluate the efficacy of dupilumab in HHD and provide solid evidence that may guide clinical practice, we performed a review of the medical literature on the use of dupilumab in the treatment of HHD. The terms “Hailey–Hailey disease”, “benign familial pemphigus”, “benign chronic pemphigus”, and “dupilumab” were searched across multiple databases (Medline, Chrocane Library, EMBASE) from inception until 30 September 2024. The literature review was carried out following PRISMA guidelines (Figure 2) [19]. Our search yielded ten results, of which one was a duplicate and was removed. The titles and abstracts of all nine remaining manuscripts were screened by two reviewers. Only reports of single cases or case series of patients with HHD treated with dupilumab were included in our study. Therefore, three articles were excluded as they represented review articles. The full texts of the six relevant publications (Table 1) [13,14,15,16,17,18] were independently assessed by two authors, who collected and analyzed the following data: the patients’ age, gender, disease duration, family history of HHD, previous treatments, dupilumab regimen, outcome, duration of dupilumab treatment, and the occurrence of adverse effects. The systematic review was not registered.

## 3. Results

To date, six manuscripts describing 11 refractory HHD cases treated with dupilumab have been published (Table 1).

**Table 1:** Cases of HHD treated with dupilumab reported in the medical literature.

Authors	Age	Gender	Disease Duration	Family History of HHD	Previous Treatments for HHD	Dupilumab Dose	Outcome	Duration of Dupilumab Treatment	Adverse Effects
Santoso et al., 2024 [13]	55	F	4 years	No	Calcipotriene, topical steroids, oral steroids, methotrexate, botulinum toxin injections, oral naltrexone	600 mg loading dose, followed by 300 mg every 2 weeks	Rapid improvement and sustained clinical remission	Sustained response 18 months after initiation of dupilumab and naltrexone 3 mg daily	None
	35	F	1 year	No	Topical tacrolimus, nystatin/triamcinolone and oral naltrexone	600 mg loading dose, followed by a 300 mg every 2 weeks	Improvement in 4 weeks and complete remission in 5 months	Sustained remission on dupilumab and naltrexone. 1.5 mg daily	None
Khang et al., 2023 [14]	53	F	25 years	No	Topical tacrolimus, oral antibiotics, topical erythromycin, and intralesional and topical steroids, oral apremilast	300-mg every 2 weeks	Marked improvement of the axillary lesions after 2 weeks, but no amelioration of the inguinal lesions. Topical ruxolitinib 1.5% cream twice a day was added, with rapid improvement and complete remission after 1 month	Sustained remission after 5 months of dupilumab and topic ruxolitinib	None
Brito Caldeira et al., 2023 [15]	57	F	Since early adulthood	No	Several topical and systemic treatments (not mentioned), including low dose naltrexone	600 mg loading dose, followed by 300 mg every 2 weeks	Symptomatic improvement after 1 week of treatment, significant healing of the skin lesions by week 12	UA	None
Licata et al., 2022 [16]	22	F	Several years	Yes (the patient’s mother)	medium-high potency topical steroids and antihistamines, oral cyclosporine	Dupilumab 300 mg every 2 weeks	Considerable improvement after 4 months	Sustained clinical remission	None
Alamon-Reig et al., 2022 [17]	56	F	10 years	No	Acitretin, antihistamines, prednisone, methotrexate, cyclosporine, hydroxychloroquine, low-dose naltrexone, apremilast, fluconazole, tetracyclines, dapsone oxybutynin, topical corticosteroids, and tacrolimus	600 mg loading dose, followed by 300 mg every 2 weeks	Significant improvement after 2 months	Clinical improvement, but not sustained complete remission during the 14-months follow-up	None
	52	M	12 years	No	Acitretin, prednisone, methotrexate, mycophenolate mofetil, cyclosporine, hydroxychloroquine, low-dose naltrexone, apremilast, fluconazole, tetracyclines, dapsone, oxybutynin, topical corticosteroids, tacrolimus, and CO2 laser ablation		Temporary clinical improvement, alternating with disease flares	Fluctuating course	None
	59	F	25 years	No	Acitretin, antihistamines, prednisone, low-dose naltrexone, apremilast, tetracyclines, dapsone, topical corticosteroids		Significant improvement after 5 months	Clinical improvement, but not sustained complete remission during the 16-months follow-up	None
Alzahrani et al., 2021 [18]	50	F	>20 years	No	Isotretinoin, etanercept, acitretin, prednisone, oral antibiotics, low dose naltrexone, cyclosporine, antihistamines, topical corticosteroids, topical clindamycin, silver sulfadiazine, clotrimazole, topical fluorouracil, various over the counter supplements	600 mg loading dose, followed by 300 mg every 2 weeks	Significant improvement after 2 months	Sustained remission for 21 months	None
	In his 50s	M	5 years	No	Topical corticosteroids, oral glycopyrrolate, acitretin, and naltrexone			Sustained remission for 25 months under dupilumab and betamethasone valerate cream;disease flare when treatment was interrupted but recaptured his response when restarted on dupilumab	None
	In his 70s	M	33 years	No	Topical therapies and injections of botulinum toxin and corticosteroids			Sustained remission for 17 months under dupilumab and desonide lotion	None

Abbreviations: F: female; M: male; UA: unavailable information.

The majority of patients were women (eight patients, 72.7%). The age of the patients ranged from 22 to 59 years, with a mean age of 50.8 ± 12.7 years. The disease duration varied widely among subjects, from 1 year to more than 30 years; the mean disease duration being 16.5 ± 11.5 years. Only one patient reported having a first-degree relative diagnosed with HHD. All the patients had previously failed at least one conventional systemic treatment and several topical treatments. In nine cases, a loading dose of 600 mg dupilumab was followed by a maintenance dose of 300 mg. In two cases, the loading dose was not administered. Concomitant treatment with low dose naltrexone was administered in two cases and topical ruxolitinib in one case. Clinical improvement was reported in all patients, and seven patients (64%) presented sustained clinical remission. However, the follow-up period varied greatly, from 5 to 24 months. In the series of three cases published by Alamon-Reig et al., considerable clinical improvement was achieved in all patients, but none experienced complete clinical remission during the 14–16 months monitoring [17]. None of the patients suffered from adverse effects related to dupilumab treatment.

Additionally, Garg et al. reported a case of refractory HHD successfully treated with tralokinumab, an anti-IL-13 biologic agent approved for the treatment of moderate–severe atopic dermatitis in adolescents and adults [2]. As tralokinumab’s mechanism of action is similar to that of dupilumab, blocking T-helper 2 cell activation, its efficacy in HHD is worth mentioning [20].

**Figure 3:**
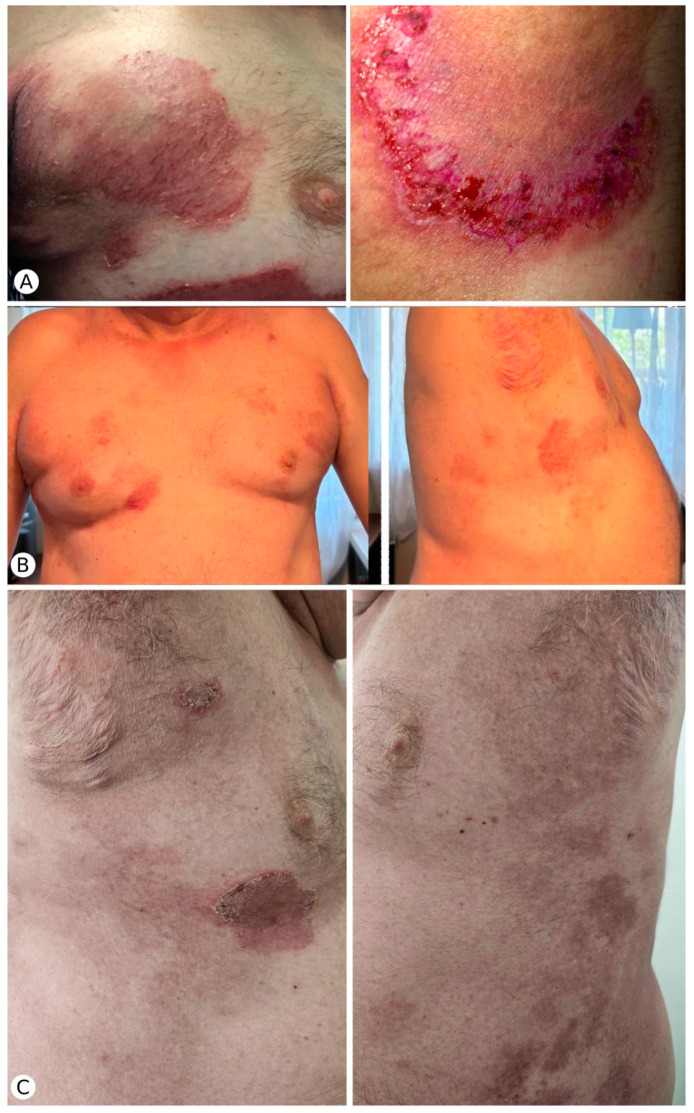
(**A**) Clinical manifestations at presentation in our clinic: extensive erythematous exudative plaques, with marked fissuring and crusted erosions affecting the inframammary folds and the axillary region, extending to the laterothoracic areas. (**B**) Significant clinical amelioration of the skin lesions 6 weeks after the initiation of dupilumab: slightly erythematous plaques with minimal fissuring located in the submammary, axillary, and laterothoracic regions. (**C**) Clinical picture 3 months after initiation of dupilumab: residual hyperpigmentation and 2 small violaceous plaques with peripheral crusting located in the right submammary and axillary areas.

We wish to add our own experience to the existing evidence by describing the case of a 57-year-old male patient who was referred to our clinic for the presence of a symmetric eruption consisting of extensive erythematous exudative plaques, with marked fissuring and crusted erosions affecting the axillary region, extending to the laterothoracic areas, inframammary folds, and inguinal and intergluteal area (Figure 3A). The patient complained of pain, burning sensation, and intense, persistent pruritus, 7/10 on the visual analogue scale (VAS), which disrupted the patient’s sleep and daily activities. He had been diagnosed with HHD in early adulthood, based on clinical and histological examination. The patient’s mother was also diagnosed with HHD, but, in her case, the onset of the condition took place at the age of 70, long after the onset of HHD in our patient. The patient’s personal and family medical history was otherwise unremarkable. Laboratory test results, including complete blood count, metabolic panel, and inflammatory markers were within normal limits.

The patient had subsequently undergone oral treatment with dapsone, retinoids, and naltrexone, associated with antiseptics, topical corticosteroids, calcineurin inhibitors, and calcipotriol. Except for naltrexone, none of the mentioned conventional systemic treatments induced clinical improvement. During the course of the illness, the patient required multiple courses of oral and parenteral corticosteroids, antibiotics, and antifungals to control severe disease flares and superinfections of the skin lesions. At presentation in our clinic, he was undergoing treatment with naltrexone 4.5 mg daily and topical treatment with antiseptics and calcipotriol. Considering the extent and severity of the disease, its refractory nature, and the major negative impact on the patient’s quality of life, off-label treatment with dupilumab was initiated in a loading dose of 600 mg, followed by a maintenance dose of 300 mg administered subcutaneously every 2 weeks. The patient’s written informed consent was obtained before treatment initiation. Treatment with naltrexone and topical antiseptics was continued.

The patient reported a marked reduction in pruritus intensity and a considerable improvement in sleep quality within 2 weeks after treatment initiation. Six weeks into dupilumab treatment, the patient presented significant clinical amelioration in the absence of new skin lesions (Figure 3B). The pruritus decreased significantly from 7/10 to 2/10 on the visual analogue scale (VAS) scale.

Almost complete clinical remission was achieved after 3 months of treatment. The patient continues treatment with dupilumab 300 mg every two weeks and naltrexone 4.5 mg daily, which are well-tolerated, with no adverse effects to date. Currently, the clinical examination only reveals residual hyperpigmentation and two small violaceous plaques with peripheral crusting located in the right inframammary and axillary areas (Figure 3C).

## 4. Discussion

As previously discussed, the causal genetic abnormality in HHD is represented by loss-of function mutations occurring throughout the ATP2C1 gene [21]. ATP2C1 is responsible for the production of a P-type secretory pathway Ca^2+^/Mn^2+^ ATPase (SPCA1), a calcium pump residing in the trans-Golgi membranes. This ubiquitous enzyme catalyzes the hydrolysis of ATP coupled with the transport of Ca^2+^ [22]. The Ca^2+^ levels in the endoplasmic reticulum, Golgi apparatus, and cytosol are finely regulated in order to ensure adequate protein synthesis [23]. Along with sarcoendoplasmic Ca^2+^–ATPase (SERCA), the normal functioning of SPCA1 is crucial for maintaining high Ca^2+^ levels in the endoplasmic reticulum and Golgi complex [23]. ATP2C1 loss-of-function mutations lead to altered Ca^2+^-dependent signaling and implicitly to perturbed protein synthesis, Golgi stress, and apoptosis [24]. SPCA1 also transports Mn^2+^ into the secretory pathway with an even higher affinity than Ca^2+^, reducing Mn^2+^ cytosolic concentrations [25]. While the toxicity of high cytosolic Mn^2+^ levels is well-known [26], its presence in the Golgi apparatus is indispensable for the optimal functioning of many enzymes. However, excessive transfer of Mn^2+^ from the cytosol into the secretory pathway is detrimental to cell survival as it binds to the same site as Ca^2+^ on SPCA1 [27], inhibiting Ca^2+^ transport and inducing Golgi stress [28].

The epidermis is the tissue most affected by ATP2C1 mutations [29]. ATP2C1 regulates the oxidative stress-induced activation of Notch1, which is essential for keratinocyte differentiation [30]. The oxidative stress associated with perturbed luminal and cytosolic Ca^2+^ homeostasis in HHD leads to altered keratinocyte differentiation and inflammation [30]. Normally, increased extrakeratinocyte Ca^2+^ concentrations activate Ca^2+^-sensing receptors and trigger actin reorganization [31]. In HHD, the dysfunctional SPCA1 fails to maintain efficient extracellular Ca^2+^ levels in the epidermis and lowers the responsiveness of Ca^2+^-sensing receptors, leading to defective Ca^2+^-sensitive actin polymerization and abnormal assembly of adherens proteins [31]. The increased cytosolic Ca^2+^ levels also trigger a decrease in ATP production [32], contributing to impaired Ca^2+^-dependent actin reorganization [31]. Additionally, the Ca^2+^ concentration in the Golgi apparatus substantially decreases, impairing the glycosylation of desmosomes [33], with subsequent impaired interkeratinocyte adhesion and suprabasal acantholysis [34].

Moreover, Ca^2+^ imbalance negatively influences keratinocyte differentiation, as it impairs the expression of keratins, especially keratin 10 and 11 [35], and the production of involucrin, a major component of the cornified envelope [36]. Normally, increased cytosolic Ca^2+^ concentrations stimulate the synthesis and transcription of involucrin mRNA [37]. Despite the characteristic high cytosolic Ca^2+^ concentrations in HHD keratinocytes, they display low involucrin levels [36]. Aberg et al. proved that the low involucrin level in HHD keratinocytes is not due to involucrin promoter activation dysfunction or altered Ca^2+^-responsiveness, nor to impaired mRNA transcription. Instead, they found that involucrin mRNA degradation is significantly more intense and rapid than in normal keratinocytes [36].

No curative treatment is available for HHD, and the primary goals of treatment are symptom control and prevention of exacerbations through suppression of inflammation and the above-mentioned general measures. Individualization of treatment is mandatory as the therapeutic response is highly variable and unpredictable.

### 4.1. Local Treatment

Topical corticosteroids represent the first line of treatment for HHD, especially at the onset of lesions. Although more effective, high potency corticosteroids should only be used during exacerbations. Topical calcineurin inhibitors can replace topical corticosteroids or be used alternately with the latter for long-term control of inflammation, especially in intertriginous areas [7,8,38].

Topical antibiotics and antiseptics assist in controlling the disease given that bacterial colonization and infection may delay the response to treatment. Good results have been obtained with clindamycin 1% cream or gel, gentamicin 0.1% cream, mupirocin 2% cream, or silver sulfadiazine cream [38].

Botulinum toxin at a dose of 50 UI per area is a valuable adjuvant treatment in HHD, reducing sweat production and inhibiting bacterial colonization [7,8,38].

Topical vitamin D analogues, such as calcitriol or calcipotriol, promote keratinocyte differentiation by regulating calcium levels, demonstrating their effectiveness when applied twice daily for long periods, usually of three months [38].

Alternative topical treatments described in isolated case reports include 5-fluorouracil cream applied three times weekly for three months and iodine cadexomer with absorbent properties [38].

In severe, refractory forms of the disease, destructive methods such as laser ablation, dermabrasion, argon plasma coagulation, cold atmospheric plasma, photodynamic therapy, and radiation therapy (electron bean or superficial radiation), as well as surgical excision of the affected area with skin grafting or secondary healing have been proposed as alternative treatments [39]. Nevertheless, HHD is often refractory to all these treatments.

### 4.2. Systemic Treatment

Antibiotics, especially those with intrinsic anti-inflammatory properties (tetracyclines, macrolides, dapsone), immunosuppressants (methotrexate, mycophenolate mofetil), oral retinoids (acitretin, alitretinoin), anticholinergics (glycopyrrolate), naltrexone, vitamin D, and magnesium chloride supplementation are therapeutic options in HHD patients, with variable efficacy [38,39].

Oral antibiotics, such as erythromycin 250 mg daily or doxycycline 100 mg daily for three months, followed by 50 mg daily, are considered second-line therapy given their anti-inflammatory and antimicrobial effects.

Naltrexone is also currently recommended as second-line treatment for HHD. It is an opioid antagonist with anti-inflammatory properties and the ability to regulate intracellular calcium levels at low doses (1.5 mg to 4.5 mg). Naltrexone exhibits paradoxical properties, including anti-inflammatory and analgesic actions. It is hypothesized that naltrexone’s action on the keratinocyte mu-opioid receptors in the basal layer of the epidermis enhances cellular adhesion and promotes the healing of skin lesions in patients with HHD. Additionally, naltrexone may antagonize toll-like receptor (TLR) complexes, which, when activated, trigger a rapid intracellular Ca²⁺ response, the nuclear translocation of nuclear factor (NF)-kB, and the production of pro-inflammatory cytokines (e.g., interleukin-1α, -1β, -8) and chemokines. These molecules initiate a pro-inflammatory response similar to that induced by microglia. By blocking these TLR complexes, naltrexone potentially reduces the downstream signaling cascade, thereby decreasing inflammation within the epidermis [4].

Oral anticholinergics, such as glycopyrrolate 1 mg daily and oxybutynin 5 mg daily, are considered treatment options due to their antiperspirant effects.

Oral retinoids regulate the epidermal keratinocyte differentiation and calcium homeostasis. Acitretin 10 to 25 mg daily or alitretinoin 30 mg daily administered for a minimum of three months are considered third-line treatments for HHD. Conversely, isotretinoin has not shown efficacy in treating HHD [40,41].

Oral corticosteroids are only indicated for the control of severe exacerbations of HHD in the short term and should be avoided whenever possible due to the frequent rebound effect.

Dapsone has both anti-inflammatory and antimicrobial properties. Numerous case reports support its use in HHD in a daily dose of 100–200 mg daily until control of the disease is achieved, followed by a maintenance dose of 50 mg daily [42].

Apremilast, a phosphodiesterase-4 inhibitor, is a low-risk alternative treatment for resistant HHD. It demonstrated efficacy in a case series of four HHD patients who were treated with 30 mg daily for one month [43,44].

Systemic immunosuppressants have been used in refractory HHD cases, although the available data on their efficacy is limited. Low dose cyclosporine (2.5 mg/kg/day) provides rapid improvement, but recurrence usually occurs after discontinuation. Nephrotoxicity and arterial hypertension may limit its use [45]. Methotrexate 7.5–15 mg weekly has been reported to induce complete remission after three months, but the rate of treatment failure is high [46]. Thalidomide 300 mg daily may be used in severe cases that are resistant to other treatments [4].

Vitamin D and magnesium chloride supplementation are therapeutic options in HHD patients, with variable efficacy [38,39].

Novel therapies, including topical and systemic Janus kinase inhibitors (upadacitinib 15 mg daily or tofacitinib 5 mg twice daily) and monoclonal antibodies (dupilumab, tralokinumab) have recently been proposed as efficient treatment options in recalcitrant HHD cases [4,14,20]. Among these, dupilumab is an especially attractive therapeutic alternative given its high efficacy in T helper (Th) 2-mediated inflammatory disorders and favorable safety profile.

The significant clinical improvement achieved in our patient adds to the evidence published so far in the medical literature regarding dupilumab’s efficacy in HHD. Several hypotheses regarding the mechanisms underlying dupilumab’s beneficial effects in HHD have been proposed.

Dupilumab inhibits Th2 mediated inflammation by blocking the type II heterodimer receptor complex shared by interleukin (IL)-4 and IL-13. These cytokines suppress SPARC-related modular calcium-binding protein 1 (SMOC1), a protein located in the basement membranes involved in intercellular adhesion during embryonic development, regulation of growth factors and keratinocyte differentiation, and maintenance of the Ca^2+^ gradient [34,47]. Therefore, dupilumab aids in the restoration of keratinocyte Ca^2+^ homeostasis and Ca^2+^ signaling [13].

IL-4 and IL-13 also modulate intracellular Ca^2+^ concentrations by activating Janus kinase (JAK) receptors, which subsequently stimulate the production of eotaxin-3 (CCL26) [18]. Eotaxin-3 receptor (CCR3) is expressed on eosinophils, basophils, and Th2 cells. Upon binding to its receptor, eotaxin-3 induces cell recruitment to inflammation sites [18,48]. Moreover, eotaxin-3 acts as an antagonist of CCR1 and CCR5, counteracting their effects, among which include inhibition of intracellular Ca^2+^ release and actin depolarization [31,49]. Thus, IL-4 and IL-13 inhibitors not only decrease cutaneous infiltration with CCR3+ inflammatory cells but also modulate intrakeratinocyte Ca^2+^ concentrations and actin depolarization, allowing normal keratinocyte differentiation and adhesion [14,20].

The onset of skin lesions in HHD usually occurs in the age group 19–65 years, which suggests a complex pathogenesis, involving both genetic and environmental exposures [27]. This might explain the variable effects of dupilumab administered in the standard regimen in the few single case reports and case series published to date. Higher dupilumab doses or shorter intervals between administrations may be considered in patients working in hot, humid environments or facing other triggering factors who experience more frequent and more severe flares.

The lack of uniform methodology of the studies that have assessed the efficacy of duplimab in the treatment of HHD hinders data analysis, impedes direct comparison of results, and impacts both theoretical advancement and practical application of the reported findings. One especially important aspect is that the monitoring period significantly differed between studies, ranging from 5 to 24 months, which raises major issues in results interpretation. The shorter follow-ups might have underestimated the long-term treatment effects and might have missed late-occurring adverse events.

## 5. Conclusions

Personalized treatment is essential for ensuring a favorable outcome in HHD given the highly variable, unpredictable response to available treatment options. Mounting evidence indicates dupilumab as a safe and efficient therapeutic alternative in patients with severe HHD, refractory to conventional treatment. The long-term efficacy of this monoclonal antibody in HHD is yet to be determined. The mechanisms underlying the efficacy of IL-4/IL-13 receptor blockers in HHD also need to be elucidated in order to optimize the therapeutic regimen.

## Data Availability

The raw data supporting the conclusions of this article will be made available by the authors on request.
